# A birth population-based survey of preterm morbidity and mortality by gestational age

**DOI:** 10.1186/s12884-021-03726-4

**Published:** 2021-04-10

**Authors:** Xiaojing Guo, Xiaoqiong Li, Tingting Qi, Zhaojun Pan, Xiaoqin Zhu, Hui Wang, Ying Dong, Hongni Yue, Bo Sun

**Affiliations:** 1 The NCH Key Laboratory of Neonatal Diseases, National Children’s Medical Center, Children’s Hospital of Fudan University, Shanghai, 201102 China; 2 Department of Obstetrics, Huai’an Women and Children’s Hospital, Huai’an, 223002 Jiangsu China; 3 Department of Neonatology and Unit of Population Health Information, Huai’an Women and Children’s Hospital, 104 South Ren Min Road, Huai’an, 223002 Jiangsu China; 4 Unit of Population Health Information, Huai’an Women and Children’s Hospital, Huai’an, 223002 Jiangsu China

**Keywords:** Birth population, Prevalence, Preterm, Morbidity, Mortality, Gestation, Regional perinatal-neonatal network

## Abstract

**Background:**

Despite 15–17 millions of annual births in China, there is a paucity of information on prevalence and outcome of preterm birth. We characterized the outcome of preterm births and hospitalized preterm infants by gestational age (GA) in Huai’an in 2015, an emerging prefectural region of China.

**Methods:**

Of 59,245 regional total births, clinical data on 2651 preterm births and 1941 hospitalized preterm neonates were extracted from Huai’an Women and Children’s Hospital (HWCH) and non-HWCH hospitals in 2018–2020. Preterm prevalence, morbidity and mortality rates were characterized and compared by hospital categories and GA spectra. Death risks of preterm births and hospitalized preterm infants in the whole region were analyzed with multivariable Poisson regression.

**Results:**

The prevalence of extreme, very, moderate, late and total preterm of the regional total births were 0.14, 0.53, 0.72, 3.08 and 4.47%, with GA-specific neonatal mortality rates being 44.4, 15.8, 3.7, 1.5 and 4.3%, respectively. There were 1025 (52.8% of whole region) preterm admissions in HWCH, with significantly lower in-hospital death rate of inborn (33 of 802, 4.1%) than out-born (23 of 223, 10.3%) infants. Compared to non-HWCH, three-fold more neonates in HWCH were under critical care with higher death rate, including most extremely preterm infants. Significantly all-death risks were found for the total preterm births in birth weight <  1000 g, GA < 32 weeks, amniotic fluid contamination, Apgar-5 min < 7, and birth defects. For the hospitalized preterm infants, significantly in-hospital death risks were found in out-born of HWCH, GA < 32 weeks, birth weight <  1000 g, Apgar-5 min < 7, birth defects, respiratory distress syndrome, necrotizing enterocolitis and ventilation, whereas born in HWCH, antenatal glucocorticoids, cesarean delivery and surfactant use decreased the death risks.

**Conclusions:**

The integrated data revealed the prevalence, GA-specific morbidity and mortality rate of total preterm births and their hospitalization, demonstrating the efficiency of leading referral center and whole regional perinatal-neonatal network in China. The concept and protocol should be validated in further studies for prevention of preterm birth.

**Supplementary Information:**

The online version contains supplementary material available at 10.1186/s12884-021-03726-4.

## Background

Perinatal and neonatal morbidity and mortality of preterm births represent the focus of quality improvement with perinatal healthcare strategies [1–5]. Despite the majority of neonatal deaths attributed to preterm birth complication, and a large amount of hospital admission-based data by gestational age (GA), available in China, the whole picture of perinatal-neonatal care remains unclear due to a lack of birth population-based clinical surveys from obstetric and neonatal perspective [6–9]. Although the number of Chinese annual birth population has reached 15–17 millions in the past decade, with an established universal health insurance covering maternal-infant healthcare [10–13], there is a paucity of data on vital statistics of preterm births and assessment of combined effects of antenatal, peripartum and postnatal interventions on perinatal and neonatal outcome in the preterm birth and hospitalized population.

Many institute-based surveys and controlled studies may provide prevalence, morbidity and mortality as outcome of hospitalized preterm population or samples, however, reliable information in birth population-based outcome of hospitalized neonatal infants remains lacking from China. For prevention of preterm birth, perinatal and neonatal death, many efforts have been exerted in the past decade with the universal maternal-infant health insurance coverage as well as facility-based robust technologies, including antenatal care with fetal ultrasound monitoring, glucocorticosteroids, tocolysis, delivery resuscitation and neonatal intensive and critical care [1–5, 7, 8, 14–16]. However, efficacy of these strategies may vary taking geographic and socioeconomic status of China into account. In this regard, further in-depth investigations are required to unravel the efficiency of perinatal-neonatal service as an integrated antenatal, peripartum and postnatal care system under particular clinical settings and infrastructure, in order to optimize facility-based practice in emerging regions in world-wide perspective.

The national surveillance system reporting on perinatal information in China has limitations as it only retrieved data of preterm births ≥28 weeks of GA [6, 17–19]. Huai’an is an emerging prefectural region in east China with 5.6-million population and 50% rural residents, the regional gross domestic production per capita approaching the national average level. According to our previous surveys of complete birth registries in Huai’an in 2015, a survival rate up to 44 weeks of adjusted postmenstrual age (aPMA) of more than 50% was observed in preterm infants with 27–28 gestational weeks and beyond [11]. By comparison, the 50% survival rate was reported in 23–25 weeks of gestation from the nation-wide registry or perinatal network data in developed countries [1–5, 7–9]. Our previous data demonstrated substantial and persistent improvement in Huai’an perinatal-neonatal care between 2010 and 2015, probably due to the establishment of centralized prenatal care and deliveries at level II and III hospitals [10, 11, 20]. This enabled us to take facility-specific conditions and regional infrastructure into consideration, when investigating the impact of integrated antenatal, peripartum and postnatal interventions on preterm especially extreme preterm survival in this region.

The current study aimed to delineate the prevalence, morbidity and mortality of whole regional preterm births and hospitalized infants stratified by GA, to investigate the role of Huai’an Women and Children’s Hospital (HWCH) as the main transferal center of whole region, and to verify the efficacy of perinatal interventions and the underlying neonatal morbidities of deaths. As birth population-based studies on perinatal and neonatal healthcare are still at preliminary stage in China [6, 12, 13], our concept and methodology of using regional birth registry data to assess death risks in hospitalized preterm population, and the quality of regional perinatal-neonatal healthcare system, may be helpful to re-evaluate and optimize the standard of care and health insurance policy in the emerging region in transition of China [6, 13].

## Methods

### Study population, protocol and ethical approval

**Fig. 1:**
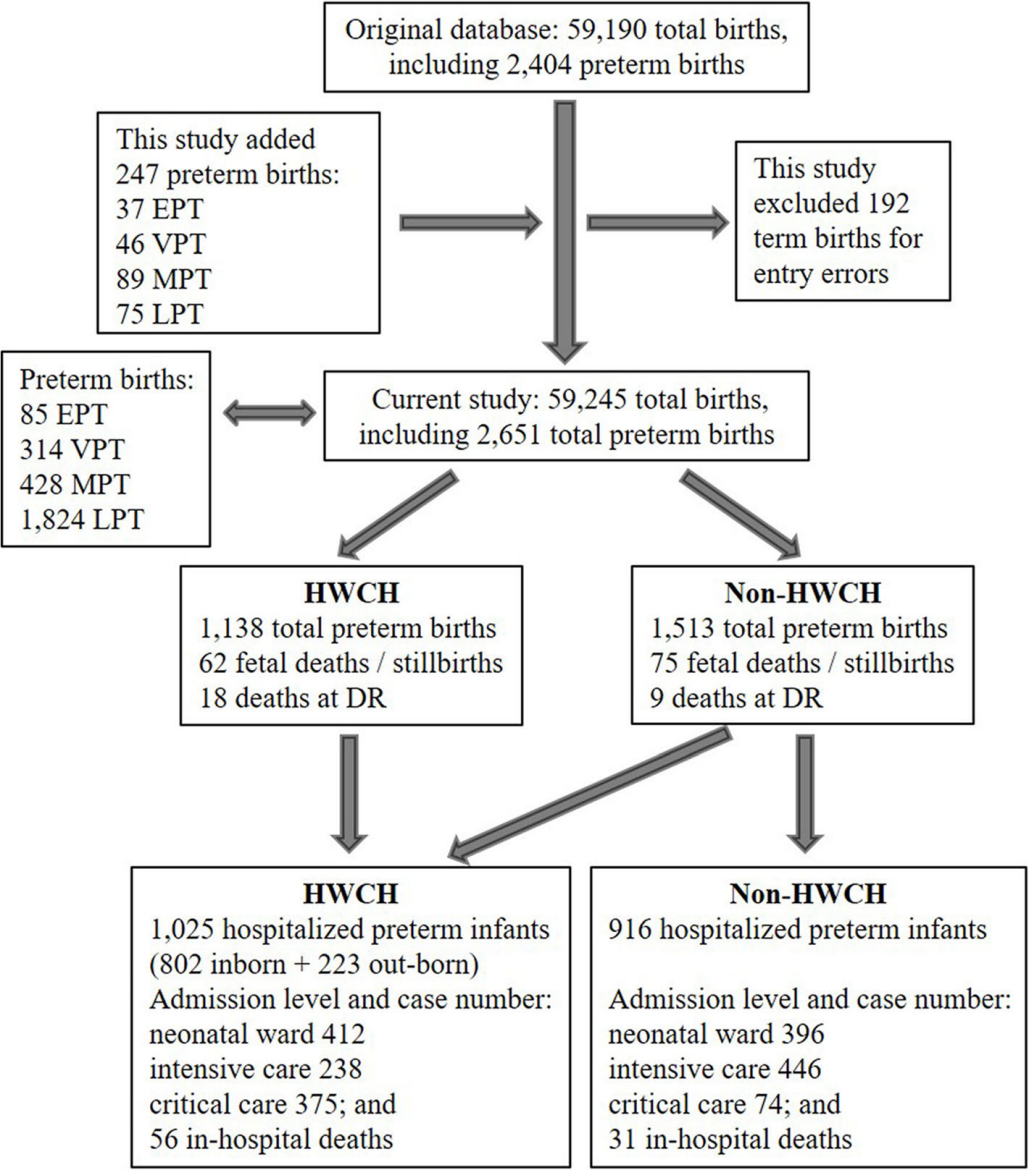
The flowchart of database and enrolled preterm population by gestational age strata and hospital categories. Abbreviations: EPT, extremely preterm; VPT, very preterm; MPT, moderate preterm; LPT, late preterm; ET, early term. For definitions of deaths and other abbreviations see Table 1 legends

The concept and protocol of investigation, definition of diagnosis of preterm morbidity and mortality as well as specific disease severity and level of care were adopted from the references cited (see below), during the data collection, analysis and preparation of manuscript in 2018–2020. This current cross-sectional study followed the whole regional birth population survey in 2015, in which a complete birth data was prospectively collected from totally 107 level I-III hospitals providing obstetric care (6 municipal, 16 county and 85 township), and 8 level II and 4 level III hospitals equipped with neonatal wards and/or intensive care units (NICU) [11, 20]. Non-medical abortions (especially unplanned pregnancy) were excluded. The above birth data was integrated with the data on all hospitalized preterm infants derived from the complete birth population in whole region, retrospectively retrieved from regional perinatal information database (Fig. 1). The study protocol was approved by the ethic committee of Children’s Hospital of Fudan University, and accepted by HWCH as well as all participating hospitals in Huai’an [11, 20]. As no specific intervention was applied, informed consent from parents/guardians was waived. Preterm birth was defined as delivery at 25^+ 0^–36^+ 6^ weeks of GA, divided into extreme (EPT, 25^+ 0^–27^+ 6^ weeks), very (VPT, 28^+ 0^–31^+ 6^ weeks), moderate (MPT, 32^+ 0^–33^+ 6^ weeks) and late (LPT, 34^+ 0^–36^+ 6^ weeks) preterm groups [21, 22]. Those of EPT below 25 weeks of GA were not included due to very few numbers and parental decision not to provide resuscitation at delivery. Incidences of EPT, VPT, MPT, LPT and total preterm births were presented as prevalence in percent of the number of total births (including term and post-term births) of whole region.

### Definitions of preterm morbidity and mortality

The diagnostic criteria of pregnancy, perinatal and neonatal co-morbidities and complications are presented in additional file 1 [23–34]. Definitions regarding vital statistics are based on the original survey [11, 20], and the 10th revision of the international classification of diseases [35]. Briefly, GA was mainly determined by the date of last menstrual period and/or fetal sonography in early pregnancy, or postnatal assessment by new Ballard score when prenatal records were missing or incomplete [36]. Birth weight (BW) was measured at birth. Small for GA (SGA) was defined as a BW < 10th percentile for gender and GA [37]. Birth defects (BD) were identified prenatally or within the first 7 postnatal days (PND) [11, 20]. Severity of neonatal underlying diseases were characterized as requiring intensive or critical care based on the treatment strength during hospitalization, such as degree of the disease severity, in need of non-invasive / mechanical ventilation (NIV/MV), surfactant, vasopressor, or surgery, etc. (also see additional file 1) [7, 28, 38]. Fetal death was deemed to be synonymous with stillbirth. Deaths at delivery room (DR) referred to those born alive but died during resuscitation, usually after parents’ requests for withdrawal or withholding [39]. Perinatal mortality included stillbirths and neonatal deaths within first 7 PND. Neonatal mortality was defined as deaths of livebirths within 28 PND, including deaths at DR. All-death was defined by including stillbirths, neonatal deaths or deaths within 44 weeks of aPMA for EPT and VPT. In-hospital death was defined as deaths during hospitalization within 28 PND, or within 44 weeks of aPMA for EPT and VPT. For those hospitalized in NICU but had early withdrawal of treatment at parental request, the outcome was estimated by attending physicians based on discharge or follow-up record.

In birth population, prevalence of perinatal and neonatal morbidities was presented as percentage (%) with the numbers of preterm total births and livebirths excluding deaths at DR as denominator (unless otherwise specified). In the hospitalized population, prevalence of postnatal morbidities was presented as percentage (%) with the number of total preterm admissions as the denominator. Rates (%) of perinatal mortality, stillbirths, deaths at DR and all-death were divided by the number of total preterm births. Neonatal mortality rate (%) was divided by the number of preterm livebirths (including deaths at DR) [20]. In-hospital death rates (%) were divided by the number of preterm livebirths excluding deaths at DR in birth population, or the number of total preterm admissions in hospitalized population, respectively. The GA-specific mortality rates (%, where numerators and denominators were both limited within specific GA stratum) [7, 40] were adopted and corrected by the number of total births or livebirths of whole region [41], and presented as per 1000 (‰, for calculation see Table 2).

### Representativeness of HWCH and risk factors of deaths

To estimate the representativeness of HWCH of whole region, whole regional preterm births and hospitalized preterm infants were divided into HWCH and non-HWCH groups, respectively. Furthermore, the HWCH admissions were divided into inborn and out-born to compare the efficacy of perinatal-neonatal care by hospital categories (Fig. 1). Risks of all-death for preterm births, and in-hospital death for preterm admissions, were analyzed to determine the role of HWCH in the whole region, taking the effects of perinatal interventions and the underlying neonatal morbidities into consideration.

### Statistical analysis

EPIDATA database was used for datasheet recordings and subjected to SPSS software (V. 16.0, SPSS Inc. Chicago, IL) for statistical analysis. Continuous variables were presented as mean and standard deviation (SD) or median [interquartile ranges]. One-way analysis of variance or non-parametric Mann-Whitney test was used for comparison among groups. Categorical variables were presented as number and rate, using a two-tailed Pearson Chi-squared or Fisher’s exact test where appropriate. *P* < 0.05 was considered statistically significant. Death risks of preterm births and hospitalizations were analyzed by uni- and multi-variable Poisson regressions by including major risk factors in terms of admission hospital categories, pregnancy complications, perinatal conditions and preterm co-morbidities as covariates. Values were given as relative risks (RR) with 95% confidence intervals (CI), or as crude and adjusted odds ratio (OR) with 95% CI for GA strata with Mantel-Haenszel Chi-square test, where appropriate.

## Results

### Preterm morbidity and mortality of whole region

**Fig. 2:**
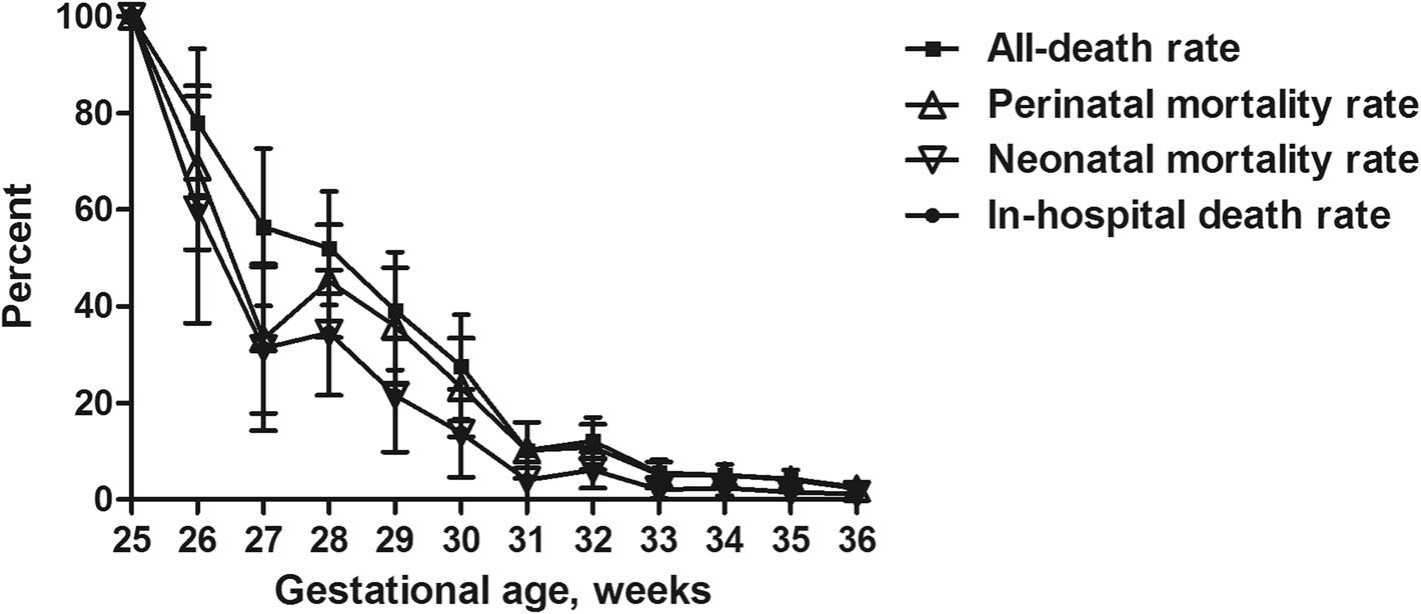
The all-death rate, perinatal mortality rate, neonatal mortality rate and in-hospital death rate and corresponding 95% confidence interval by each gestational age. Legends: For definitions of death rates, see Table 1 footnotes

Of the 59,245 regional total births (and 59,056 livebirths), 2651 (4.5%) were preterm in which 2527 women had fetal sonography early in pregnancy, with preterm perinatal and neonatal mortality rates being 8.4 and 4.3%, respectively (Table 1). The prevalence of EPT, VPT, MPT, LPT and total preterm of the total births of whole region were 0.14, 0.53, 0.72, 3.08 and 4.47%, with corresponding all-death rate 1.0, 1.6, 0.6, 1.1 and 4.3‰, respectively (Table 2), and GA-specific neonatal mortality rate 44.4, 15.8, 3.7, 1.5 and 4.3%, respectively. The GA-specific all-death rates of 25, 26, 27 and 28 weeks were 100, 78.1, 56.4 and 52.1%, and in-hospital death rates 100, 70.0, 48.3 and 30.4%, with a 50% survival rate achieved by 28 and 27 week of GA, respectively (Fig. 2).

**Table 1:** Perinatal demographic status, prevalences and mortality rates of preterm births in Huai’an in 2015

	HWCH	Non-HWCH	Whole region
Total births	9405	49,840	59,245
Total live births	9328	49,728	59,056
Preterm births^1^***	1138 (12.1)	1513 (3.0)	2651 (4.5)
Preterm livebirths	1076 (94.6)	1438 (95.0)	2514 (94.8)
HDP***	253 (22.2)	182 (12.0)	435 (16.4)
GDM***	102 (9.0)	21 (1.4)	123 (4.6)
Anemia***	287 (25.2)	88 (5.8)	375 (14.1)
PROM	377 (33.1)	446 (29.5)	823 (31.0)
ANG**	513 (45.1)	596 (39.4)	1109 (41.8)
Cesarean delivery*	685 (60.2)	851 (56.2)	1536 (57.9)
Male	649 (57.0)	865 (57.2)	1514 (57.1)
GA, weeks**	33.8 + 2.8	34.1 + 2.3	34.0 + 2.5
25–27***	61 (5.4)	24 (1.6)	85 (3.2)
28–31	132 (11.6)	182 (12.0)	314 (11.8)
32–33	173 (15.2)	255 (16.9)	428 (16.1)
34–36	772 (67.8)	1052 (69.5)	1824 (68.8)
BW, g	2369 + 656	2420 + 657	2398 + 657
< 1000***	57 (5.0)	12 (0.8)	69 (2.6)
1000-1499	85 (7.5)	118 (7.8)	203 (7.7)
1500-2499	467 (41.0)	616 (40.7)	1083 (40.9)
> 2500*	529 (46.5)	767 (50.7)	1296 (48.9)
Multiple births***	311 (27.3)	294 (19.4)	601 (22.8)
SGA	47 (4.1)	70 (4.6)	117 (4.4)
AF contamination*	72 (6.3)	128 (8.5)	200 (7.5)
Grade II	8 (0.7)	27 (1.8)	35 (1.3)
Grade III	48 (4.2)	50 (3.3)	98 (3.7)
Apgar 1-min < 7***	472 (41.5)	278 (18.4)	750 (28.3)
Apgar 5-min < 7***	175 (15.4)	156 (10.3)	331 (12.5)
Birth defects	36 (3.2)	45 (3.0)	81 (3.1)
Hospitalization^2^*	802 (75.8)	1139 (79.7)	1941 (78.0)
Perinatal mortality^3^	101 (8.9)	123 (8.1)	224 (8.4)
Fetal deaths/stillbirths	62 (5.4)	75 (5.0)	137 (5.2)
Deaths at DR*	18 (1.6)	9 (0.7)	27 (1.0)
Neonatal mortality^4^	49 (4.6)	58 (4.0)	107 (4.3)
In-hospital deaths^5^	33 (3.1)	54 (3.8)	87 (3.5)
All-death^6^	114 (10.0)	138 (9.1)	252 (9.5)

Abbreviations: HWCH, Huai’an Women and Children’s Hospital; non-HWCH, hospitals excluding HWCH; HDP, hypertensive disorder of pregnancy; GDM, gestational diabetes  mellitus; PROM, prelabor rupture of membrane; ANG, antenatal glucocorticoids; GA, gestational age; BW, birth weight; SGA, small for gestational age; AF, amniotic fluid; DR, delivery room

Values are given in n, n (%) or means + SD. Percentage (%) refers to number of preterm births in respective columns unless otherwise stated. * *P* < 0.05, ** *P* < 0.01 and *** *P* < 0.001 for HWCH vs. non-HWCH

1. Percentage (%) refers to the number of total births (including term and post term births) in respective columns

2. Percentage (%) refers to number of preterm livebirths excluding deaths at DR in respective columns

3. Perinatal mortality includes preterm fetal deaths / stillbirths from 25 complete gestational week till deaths within the first 7 postnatal days (including deaths at DR)

4. Neonatal mortality includes preterm deaths at DR and of livebirths within 28 postnatal days. Percentage (%) refers to number of preterm livebirths in respective columns

5. In-hospital deaths include preterm deaths of hospitalized infants until 44 week of adjusted post menstrual age (excluding deaths at DR). Percentage (%) refers to above note 2 for definition

6. All-death includes preterm fetal deaths / stillbirths and postnatal deaths from 25 complete gestational week till 44 week of adjusted post menstrual age

**Table 2:** Constitution of GA-specific mortality rates or corrected by the total births of whole region

GA, weeks	25–27	28–31	32–33	34–36	25–36
Preterm births, n	85	314	428	1824	2651
Prevalence, ‰^1^	1.4	5.3	7.2	30.8	44.7
Perinatal mortality, n	49	83	32	60	224
R1, %	57.6	26.4	7.5	3.3	8.4
R2, ‰	0.8	1.4	0.5	1.0	3.8
Fetal deaths / stillbirths, n	31	49	20	37	137
R1, %	36.5	15.6	4.7	2.0	5.2
R2, ‰	0.5	0.8	0.3	0.6	2.3
Deaths at DR, n	12	11	2	2	27
R1, %	14.1	3.5	0.5	0.1	1.0
R2, ‰	0.2	0.2	0.0	0.0	0.5
Neonatal mortality, n	24	42	15	26	107
R1, %^2^	44.4	15.8	3.7	1.5	4.3
R2, ‰^3^	0.4	0.7	0.3	0.4	1.8
All deaths, n	61	93	35	63	252
R1, %	71.8	29.6	8.2	3.5	9.5
R2, ‰	1.0	1.6	0.6	1.1	4.3

Abbreviations: GA, gestational age; DR, delivery room. Values are number (n) or rate. GA-specific mortality rate (R1, %) = (number of deaths of each GA stratum / number of births of the same GA stratum) × 100; GA-specific mortality rate corrected by the number of total births (including term and post term births) of whole region (R2, ‰) = GA-specific mortality rate × prevalence of each GA in the total births = (number of deaths of each GA stratum / the number of total births [59,245]) × 1000. For definition of all-death see Table 1 legends, note 6

1. Prevalences of each GA in the total births (‰) = (number of births of each GA stratum / the number of total births) × 1000

2. R1, calculated by number of neonatal deaths of each GA stratum divided by number of livebirths in the same GA stratum

3. R2, calculated by number of neonatal deaths of each GA stratum divided by the number of livebirths (including term and post term births) of whole region (59,056)

Of 2487 preterm livebirths excluding deaths at DR, the hospitalization and in-hospital death rate was 78.0 and 3.5%, respectively (Table 1). In 1941 hospitalized preterm infants (68 had GA determined by new Ballard score), 35.2 and 23.1% received intensive and critical care (Table 3), with in-hospital death rates being 0.6% (4/684) and 18.5% (83/449), respectively. The top five morbidities in hospitalized preterm infants were hyperbilirubinemia (59.0%), pneumonia/sepsis (40.0%), respiratory distress syndrome (RDS) (14.1%), intraventricular hemorrhage (11.0%) and hypoglycemia (10.8%), respectively. Surfactant and NIV/MV were applied in 11.2 and 39.9% of the hospitalized preterm infants, respectively (Table 3).

**Table 3:** Perinatal status, morbidity and mortality rates of all hospitalized preterm infants in Huai’an

	HWCH			Non-HWCH	Whole
	Inborn	Out-born	Total	hospitals	region
Hospitalization	802	223	1025	916	1941
Intensive care	184 (22.9)	54 (24.2)	238 (23.2)	446 (48.7)###	684 (35.2)
Critical care^1^	283 (35.3)	92 (41.3)	375 (36.6)	74 (8.1)###	449 (23.1)
Length of stay, days	14 [10, 19]	14 [9, 21]	14 [10, 20]	8 [5, 13]###	11 [7, 17]
Costs, ×1000 CNY	13 [9, 20]	13 [9, 23]	13 [9, 20]	7 [4, 12]###	10 [6, 17]
Cesarean delivery	509 (63.5)	104 (46.6)***	613 (59.8)	556 (60.7)	1169 (60.2)
Male	472 (58.9)	127 (57.0)	599 (58.4)	496 (54.1)	1095 (56.4)
GA, weeks	33.7 + 2.4	33.6 + 2.5	33.7 + 2.4	34.1 + 2.1###	33.9 + 2.3
25–27	21 (2.6)	7 (3.1)	28 (2.7)	12 (1.3)#	40 (2.1)
28–31	109 (13.6)	34 (15.2)	143 (14.0)	98 (10.7)	241 (12.4)
32–33	160 (20.0)	44 (19.7)	204 (19.9)	155 (16.9)	359 (18.5)
34–36	512 (63.8)	138 (61.9)	650 (63.4)	651 (71.1)##	1301 (67.0)
BW, g	2280 + 610	2270 + 625	2278 + 614	2376 + 636##	2324 + 626
< 1000	12 (1.5)	1 (0.4)	13 (1.3)	7 (0.8)	20 (1.0)
1000-1499	72 (9.0)	27 (12.1)	99 (9.7)	73 (8.0)	172 (8.9)
1500-2499	403 (50.2)	110 (49.3)	513 (50.0)	422 (46.1)	935 (48.2)
> 2500	315 (39.3)	85 (38.1)	400 (39.0)	414 (45.2)##	814 (41.9)
Multiple births	214 (26.7)	35 (15.7)**	249 (24.3)	140 (15.0)###	389 (20.0)
SGA	35 (4.4)	12 (5.4)	47 (4.6)	50 (5.5)	97 (5.0)
AF contamination	39 (4.9)	15 (6.7)	54 (5.3)	66 (7.2)	120 (6.2)
Grade II	5 (0.6)	4 (1.8)	9 (0.9)	17 (1.9)	26 (1.3)
Grade III	24 (3.0)	9 (4.0)	33 (3.2)	26 (2.8)	59 (3.0)
Apgar 1-min < 7	375 (46.8)	42 (18.8)***	417 (40.7)	156 (17.0)###	573 (29.5)
Apgar 5-min < 7	91 (11.3)	24 (10.8)	115 (11.2)	52 (5.7)###	167 (8.6)
Birth defects	26 (3.1)	20 (9.0)***	45 (4.4)	109 (11.9)###	154 (7.9)
Admitted within 24 h	711 (88.7)	145 (65.0)***	856 (83.5)	712 (77.7)##	1568 (80.8)
IVH	99 (12.3)	24 (10.8)	123 (12.0)	90 (9.8)	213 (11.0)
RDS	119 (14.8)	38 (17.0)	157 (15.3)	116 (12.7)	273 (14.1)
Pneumonia / sepsis	367 (45.8)	116 (52.0)	483 (47.1)	294 (32.1)###	777 (40.0)
Hypoglycemia	148 (18.5)	31 (13.9)	179 (17.5)	30 (3.3)###	209 (10.8)
PDA	11 (1.4)	4 (1.8)	15 (1.5)	4 (0.4)#	19 (1.0)
Hyperbilirubinemia	690 (86.0)	118 (52.9)***	808 (78.8)	338 (36.9)###	1146 (59.0)
Air leaking	3 (0.4)	4 (1.8)*	7 (0.7)	3 (0.3)	10 (0.5)
Surfactant	103 (12.8)	28 (12.6)	131 (12.8)	86 (9.4)#	217 (11.2)
NIV / MV	309 (38.5)	95 (42.6)	404 (39.4)	370 (40.4)	774 (39.9)
BPD	32 (4.0)	8 (3.6)	40 (3.9)	13 (1.4)##	53 (2.7)
NEC	21 (2.6)	7 (3.1)	28 (2.7)	10 (1.1)##	38 (2.0)
ROP	6 (0.7)	0*	6 (0.6)	0#	6 (0.3)
Encephalopathy	33 (4.1)	10 (4.5)	43 (4.2)	15 (1.6)##	58 (3.0)
In-hospital deaths	33 (4.1)	23 (10.3)***	56 (5.5)	31 (3.4)#	87 (4.5)
By postnatal days					
0–6	20 (2.5)	15 (6.7)**	35 (3.4)	22 (2.4)	57 (2.9)
7–27	10 (1.2)	5 (2.2)	15 (1.5)	8 (0.9)#	23 (1.2)
> 28	3 (0.4)	3 (1.3)	6 (0.6)	1 (0.1)	7 (0.4)
By GA weeks					
25–27	8 (1.0)	5 (2.2)	13 (1.3)	9 (1.0)	22 (1.1)
28–31	15 (1.9)	10 (4.5)*	25 (2.4)	13 (1.4)	38 (2.0)
32–33	6 (0.7)	3 (1.3)	9 (0.9)	3 (0.3)	12 (0.6)
34–36	4 (0.5)	5 (2.2)*	9 (0.9)	6 (0.7)	15 (0.8)

Abbreviations: CNY, Chinese Yuan (RMB); IVH, intraventricular hemorrhage; RDS, respiratory distress syndrome; PDA, patent ductus arteriosus; NIV / MV, non-invasive ventilation / mechanical ventilation; BPD, bronchopulmonary dysplasia; NEC, necrotizing enterocolitis; ROP, retinopathy of prematurity. Values are given as n, n (%), means + SD or median [interquartile ranges]. Percentage (%) refers to number of total hospitalization in respective columns. * *P* < 0.05, ** *P* < 0.01 and *** *P* < 0.001 for out-born vs. inborn of HWCH. # *P* < 0.05, ## *P* < 0.01 and ### *P* < 0.001 for non-HWCH vs. total of HWCH. For other definitions and abbreviations see Table 1 legends as reference

1. For definition of critical care see additional file 1

### Representativeness of HWCH

In the 59,245 total births of whole region, 9405 (15.9%) were born in HWCH, with significantly higher rates of major pregnancy complications and preterm birth (> three-fold) than those of non-HWCH hospitals (Table 1). Of the whole region, proportions of births and all-deaths for EPT in HWCH were 71.8 and 78.7%, respectively. A total of 1025 (52.8% of whole region) preterm infants were hospitalized in HWCH (802 inborn and 223 out-born). The in-hospital death rate was significantly higher in the out-born than in the inborn infants, especially within 7 PND, and in VPT and LPT (Table 3). Compared to non-HWCH, the rate of hospitalized preterm infants received critical care was four times as high as in HWCH, with significantly longer length of stay, higher costs, and higher rates of in-hospital deaths and major morbidities (Table 3).

### Uni- and multi-variable Poisson regression analysis

In general, for the variables with moderate to high relative risks of deaths by univariable regression analysis, it tended to become mild or no risk by multivariable regression model. The effect of mitigation of risks was also seen in stratified analysis by GA (Table S1). Significantly independent all-death risks were found for the total preterm births in GA < 32 weeks, BW <  1000 g, amniotic fluid contamination, Apgar-5 min < 7, and BD (Table 4a). For the hospitalized preterm infants, significantly in-hospital death risks were found in the out-born of HWCH, GA < 32 weeks, BW <  1000 g, Apgar-5 min < 7, BD, RDS, necrotizing enterocolitis and NIV/MV (Table 4b), whereas born in HWCH, antenatal glucocorticoids, cesarean delivery and surfactant use reduced death risks (Table 4a and b).

**Table 4:** Uni- and multivariable Poisson regression analysis of death risks of the whole Huai’an region

A. The total preterm births				
Variables	Category	All-death n (%)	Univariable RR (95% CI)	Multivariable RR (95% CI)
Born in HWCH	No	138 (9.1)		
	Yes	114 (10.0)	1.10 (0.87–1.39)	0.65 (0.52–0.82)^3^
PROM	No	192 (10.5)		
	Yes	60 (7.3)	0.69 (0.53–0.92)^1^	1.09 (0.88–1.35)
HDP	No	215 (9.7)		
	Yes	37 (8.5)	0.88 (0.63–1.22)	1.13 (0.86–1.48)
GDM	No	239 (9.5)		
	Yes	13 (10.6)	1.12 (0.66–1.90)	1.39 (0.92–2.12)
Anemia	No	228 (10.0)		
	Yes	24 (6.4)	0.64 (0.43–0.96)^1^	0.84 (0.63–1.12)
ANG	No	182 (11.8)		
	Yes	70 (6.3)	0.54 (0.41–0.70)^3^	0.49 (0.27–0.74)^1^
Cesarean delivery	No	186 (16.7)		
	Yes	66 (4.3)	0.26 (0.20–0.34)^3^	0.73 (0.56–0.96)^1^
Male	No	110 (9.7)		
	Yes	140 (9.2)	0.95 (0.75–1.21)	0.96 (0.81–1.15)
GA, weeks	34–36	63 (3.5)		
	32–33	35 (8.2)	2.37 (1.59–3.53)^3^	1.15 (0.74–1.77)
	28–31	93 (29.6)	8.58 (6.38–11.5)^3^	1.66 (1.10–2.51)^1^
	25–27	61 (71.8)	20.8 (15.8–27.4)^3^	1.67 (1.02–2.72)^1^
BW, g	> 2500	43 (3.3)		
	1500-2499	81 (7.5)	2.25 (1.57–3.23)^3^	0.96 (0.65–1.41)
	1000-1499	63 (31.0)	9.35 (6.54–13.4)^3^	1.23 (0.77–1.95)
	< 1000	65 (94.2)	28.4 (21.0–38.3)^3^	2.25 (1.38–3.67)^2^
Multiple births	No	205 (10.0)		
	Yes	47 (7.8)	0.78 (0.57–1.05)	0.98 (0.78–1.25)
SGA	No	199 (7.9)		
	Yes	24 (20.5)	2.58 (1.76–3.78)^3^	1.09 (0.79–1.51)
AF contamination	No	191 (7.8)		
	Yes	61 (30.5)	3.91 (3.05–5.02)^3^	1.46 (1.16–1.84)^2^
5-min Apgar < 7	No	49 (2.1)		
	Yes	203 (61.3)	29.0 (21.7–38.8)^3^	14.7 (9.63–22.5)^3^
Birth defects	No	219 (8.5)		
	Yes	33 (40.7)	4.78 (3.57–6.40)^3^	1.73 (1.28–2.33)^3^
**B. The hospitalized preterm infants**				
Variables	Category	In-hospital deaths, n (%)	Univariable RR (95% CI)	Multivariable RR (95% CI)
Admitted in HWCH	No	31 (3.4)		
	Inborn	33 (4.1)	1.22 (0.75–1.97)	0.96 (0.58–1.58)
	Out-born	23 (10.3)	3.05 (1.81–5.12)^3^	2.27 (1.40–3.69)^2^
Male	No	48 (5.7)		
	Yes	39 (3.6)	0.63 (0.42–0.95)^1^	0.73 (0.50–1.07)
GA, weeks	34–36	15 (1.2)		
	32–33	12 (3.3)	2.90 (1.37–6.14)^2^	1.49 (0.68–3.26)
	28–31	38 (15.8)	13.7 (7.65–24.5)^3^	2.31 (1.02–5.26)^1^
	25–27	22 (55.0)	47.7 (26.8–84.9)^3^	2.44 (0.90–6.61)
BW, g	> 2500	9 (1.1)		
	1500-2499	23 (2.5)	2.23 (1.04–4.78)^1^	0.95 (0.40–2.25)
	1000-1499	41 (23.8)	21.6 (10.7–43.5)^3^	2.25 (0.83–6.13)
	< 1000	14 (70.0)	63.3 (31.1–128)^3^	3.66 (1.16–11.6)^1^
Cesarean delivery	No	58 (7.5)		
	Yes	29 (2.5)	0.33 (0.21–0.51)^3^	0.53 (0.34–0.83)^2^
5-min Apgar < 7	No	47 (2.6)		
	Yes	40 (24.0)	9.04 (6.12–13.4)^3^	2.73 (1.75–4.26)^3^
Birth defects	No	67 (3.7)		
	Yes	20 (13.0)	3.46 (2.16–5.55)^3^	2.57 (1.59–4.16)^3^
Admitted within 24 h (after birth)	No	8 (2.1)		
	Yes	79 (5.0)	2.35 (1.15–4.82)^1^	0.81 (0.39–1.69)
RDS	No	29 (1.7)		
	Yes	58 (21.2)	12.2 (7.97–18.7)^3^	2.76 (1.65–4.65)^3^
Pneumonia/sepsis	No	20 (1.7)		
	Yes	67 (8.6)	5.02 (3.07–8.20)^3^	0.74 (0.43–1.28)
NEC	No	72 (3.8)		
	Yes	15 (39.5)	10.4 (6.62–16.4)^3^	1.76 (1.03–3.03)^1^
Surfactant	No	50 (2.9)		
	Yes	37 (17.1)	5.88 (3.94–8.78)^3^	0.43 (0.28–0.66)^3^
NIV/MV	No	6 (0.5)		
	Yes	81 (10.5)	20.4 (8.93–46.4)^3^	6.69 (2.07–21.7)^2^

Values are presented as number of deaths (%) and corresponding relative risk (RR) and its 95% confidence intervals (95% CI) by Poisson regression analysis. Multivariable analysis was done by all listed factors in the first column. For definition of all-death see Table 1 legends, note 6; for definition of in-hospital deaths see Table 1 legends, note 5; for other definitions and abbreviations see Tables 1 and 3 legends as reference

1–3, and stands for *P* < 0.05, *P* < 0.01 and *P* < 0.001, respectively

## Discussions

Our study demonstrated the prevalences, perinatal and neonatal morbidity and mortality by GA strata of total preterm births or hospitalized preterm infants, from a complete birth population in an emerging region of China. These findings should be complementary to the findings from reports of national surveys by sampling, or institution-based studies on perinatal and neonatal outcome in China, which lacked comprehensive perinatal data from the regional birth-population perspective [42–45]. As a result, we speculate that our understanding on the preterm birth status in China, either nation-wide or province-wide, would have substantial negligence, error and bias in terms of source and representativeness of data and database. We assumed that the Huai’an region represented the national average level of socioeconomic and healthcare status, and it may reflect the actual perinatal-neonatal healthcare style and quality in one-fourth of sub-provincial prefectural regions in China. Our data showed approximately 4.5% of preterm birth rate and 10% preterm mortality rate. This may be translated into annually 650,000 preterm births and 65,000 deaths in China. The concept, methodology and data file of the current study may be generalizable to other regions, and served as a benchmark for future large-scale studies to explore perinatal-neonatal healthcare quality improvement, especially in the care of VPT and EPT population.

The prevalence of EPT (25–27 weeks of GA, 1.4‰ of total births, Table 2) was first reported from birth population-based survey data from China [11, 20], and was markedly lower than the 3–5‰ of total births in developed countries [3–9]. Despite the benefits provided by the universal health insurance policy for hospitalized delivery, especially for families with lower socioeconomic status, it remains controversial whether active prenatal and peripartum management including aggressive resuscitation at delivery, and neonatal critical care, should be provided to EPT in emerging regions in China. As deaths in livebirths below 28-week GA may be registered as abortion or stillbirth [19], or as neonatal death only after 7th of PND (unpublished domestic recommendation for birth registry since 2018), it can cause potential bias on vital statistics of the incidence/prevalence and outcome of preterm births and mortalities [7, 17, 18], leading to inaccurate estimation of the quality improvement of perinatal-neonatal care.

The comparison of preterm outcome between HWCH and non-HWCH admissions shed light on the role of main transferal centers in regional perinatal-neonatal network. As revealed in Tables 1 and 3, HWCH treated a disproportionally large part of high-risk pregnancies in the Huai’an region [20], and admitted more extremely and very preterm infants requiring critical care than other regional institutions. It implies a generous and effective in-utero transport policy taking place. It also supports a central role of HWCH in the regional perinatal-neonatal healthcare, and indicates that investigation of the leading centers may help to understand the perinatal-neonatal healthcare at the regional level. Of note, the vast sub-provincial prefectural regions in China are estimated to account for more than 80% of annual national births (of 15–17 millions in 2014–2019). The inter-institutional difference in infrastructure should be narrowed with recent socioeconomic development, but quality of maternal-neonatal healthcare remains to vary widely. By including all birth data from level I-III institutions in the whole region of Huai’an [11, 20], our study has overcome the limitations encountered in previous reports of vital statistics which often failed to account for prevalence and outcome of very and extreme preterm births [6, 17–19]. Based on the earlier nationwide collaborative studies, the quality of perinatal-neonatal healthcare at HWCH is likely to be above the national average level [11, 20, 42, 43] and the data file of outcome may be referred to for future inter-regional comparison to validate.

There was a trend that those with high or moderate risk of death by univariable regression analysis tended to have mild or no risk by multivariable Poisson regression model (Table 4), well denoting that with decent obstetric management for high risk pregnancy and delivery, as well as NICU service quality, death risks of preterm birth and hospitalization may be effectively mitigated. As estimated by relative death risks of preterm birth and hospitalization through multivariable Poisson regression model, these should have explained, at least in part, the discrepancy of GA-specific mortality rates between birth and hospitalized population [7–11]. We therefore speculate that it should enable a comprehensive assessment of quality improvement in the regional perinatal-neonatal healthcare infrastructure as well as survival quality in follow-up of very and extremely preterm infants [1–5, 8, 9].

The main limitation of the study was a relatively small sample size of EPT and VPT. However, as the first report of outcome of all hospitalized preterm infants derived from the total regional birth population, this study, on the other hand, has accounted for a large number of perinatal and postnatal risk factors as composite effects on the outcome measures from preterm birth to the whole hospitalization. Another limitation of the study was that no details of peripartum intervention were included for the perinatal risk analysis. Nevertheless, information on the prevalence of preterm births, morbidity and mortality by GA and efficacy of perinatal interventions may represent to a large part as a baseline of the regional perinatal-neonatal outcome, given the proportion of preterm infants requiring critical care and out-born infants transferal at HWCH and other level III hospitals.

## Conclusion

In conclusion, the prevalence of preterm birth and hospitalization based morbidity and mortality by GA strata or hospital categories, as characterized for both HWCH and non-HWCH, reflected the baseline and quality of healthcare status of regional network, representing the perinatal-neonatal healthcare in emerging regions. The concept and protocol of the study should be feasible to other regions of China and beyond to gain comprehensive understanding in the world-wide campaign for prevention of preterm birth.

## Supplementary Information

**Additional file 1.** Definitions of pregnancy and perinatal co-morbidities and complications and standard of care at admission and during hospitalization of preterm infants.

**Additional file 2.** A full list of members and their affiliations of Huai’an Perinatal-Neonatal study group.

**Additional file 3: Table S1.** Crude and gestational age-adjusted odds ratio (OR) of death risks of the whole Huai’an region.

## Data Availability

The datasets used and/or analyzed during the current study are available from the corresponding author on reasonable request.

## Abbreviations

aPMA: adjusted post menstrual age
BD: birth defects
BPD: bronchopulmonary dysplasia
BW: birth weight
DR: delivery room
EPT: extreme preterm
GA: gestational age
HWCH: Huai’an Women and Children’s Hospital
LPT: late preterm
MPT: moderate preterm
MV: mechanical ventilation
NICU: neonatal intensive care unit
NIV: non-invasive ventilation
PND: postnatal day(s)
RDS: respiratory distress syndrome
VPT: very preterm
