# Correction to: A birth population-based survey of preterm morbidity and mortality by gestational age

**DOI:** 10.1186/s12884-021-03841-2

**Published:** 2021-05-12

**Authors:** Xiaojing Guo, Xiaoqiong Li, Tingting Qi, Zhaojun Pan, Xiaoqin Zhu, Hui Wang, Ying Dong, Hongni Yue, Bo Sun

**Affiliations:** 1 The NCH Key Laboratory of Neonatal Diseases, National Children’s Medical Center, Children’s Hospital of Fudan University, Shanghai, 201102 China; 2 Department of Obstetrics, Huai’an Women and Children’s Hospital, Huai’an, 223002 Jiangsu China; 3 Department of Neonatology and Unit of PopulationHealth Information, Huai’an Women and Children’s Hospital, 104 South Ren Min Road, Huai’an, 223002 Jiangsu China; 4 Unit of Population Health Information, Huai’an Women and Children’s Hospital, Huai’an, 223002 Jiangsu China

**Correction to: BMC Pregnancy Childbirth 21, 291 (2021)**

**https://doi.org/10.1186/s12884-021-03726-4**

Following publication of the original article [1], the authors reported an error in Table 4 heading (continued) in the PDF version is incorrect, “A. The total preterm births” should be “B. The hospitalized preterm infants”.

**Table 4:** Uni- and multivariable Poisson regression analysis of death risks of the whole Huai’an region

**A. The total preterm births**				
Variables	Category	All-death n (%)	Univariable RR (95% CI)	Multivariable RR (95% CI)
Born in HWCH	No	138 (9.1)		
	Yes	114 (10.0)	1.10 (0.87–1.39)	0.65 (0.52–0.82)^3^
PROM	No	192 (10.5)		
	Yes	60 (7.3)	0.69 (0.53–0.92)^1^	1.09 (0.88–1.35)
HDP	No	215 (9.7)		
	Yes	37 (8.5)	0.88 (0.63–1.22)	1.13 (0.86–1.48)
GDM	No	239 (9.5)		
	Yes	13 (10.6)	1.12 (0.66–1.90)	1.39 (0.92–2.12)
Anemia	No	228 (10.0)		
	Yes	24 (6.4)	0.64 (0.43–0.96)^1^	0.84 (0.63–1.12)
ANG	No	182 (11.8)		
	Yes	70 (6.3)	0.54 (0.41–0.70)^3^	0.49 (0.27–0.74)^1^
Cesarean delivery	No	186 (16.7)		
	Yes	66 (4.3)	0.26 (0.20–0.34)^3^	0.73 (0.56–0.96)^1^
Male	No	110 (9.7)		
	Yes	140 (9.2)	0.95 (0.75–1.21)	0.96 (0.81–1.15)
GA, weeks	34–36	63 (3.5)		
	32–33	35 (8.2)	2.37 (1.59–3.53)^3^	1.15 (0.74–1.77)
	28–31	93 (29.6)	8.58 (6.38–11.5)^3^	1.66 (1.10–2.51)^1^
	25–27	61 (71.8)	20.8 (15.8–27.4)^3^	1.67 (1.02–2.72)^1^
BW, g	> 2500	43 (3.3)		
	1500-2499	81 (7.5)	2.25 (1.57–3.23)^3^	0.96 (0.65–1.41)
	1000-1499	63 (31.0)	9.35 (6.54–13.4)^3^	1.23 (0.77–1.95)
	< 1000	65 (94.2)	28.4 (21.0–38.3)^3^	2.25 (1.38–3.67)^2^
Multiple births	No	205 (10.0)		
	Yes	47 (7.8)	0.78 (0.57–1.05)	0.98 (0.78–1.25)
SGA	No	199 (7.9)		
	Yes	24 (20.5)	2.58 (1.76–3.78)^3^	1.09 (0.79–1.51)
AF contamination	No	191 (7.8)		
	Yes	61 (30.5)	3.91 (3.05–5.02)^3^	1.46 (1.16–1.84)^2^
5-min Apgar < 7	No	49 (2.1)		
	Yes	203 (61.3)	29.0 (21.7–38.8)^3^	14.7 (9.63–22.5)^3^
Birth defects	No	219 (8.5)		
	Yes	33 (40.7)	4.78 (3.57–6.40)^3^	1.73 (1.28–2.33)^3^
**B. The hospitalized preterm infants**				
Variables	Category	In-hospital deaths n (%)	Univariable RR (95% CI)	Multivariable RR (95% CI)
Admitted in HWCH	No	31 (3.4)		
	Inborn	33 (4.1)	1.22 (0.75–1.97)	0.96 (0.58–1.58)
	Out-born	23 (10.3)	3.05 (1.81–5.12)^3^	2.27 (1.40–3.69)^2^
Male	No	48 (5.7)		
	Yes	39 (3.6)	0.63 (0.42–0.95)^1^	0.73 (0.50–1.07)
GA, weeks	34–36	15 (1.2)		
	32–33	12 (3.3)	2.90 (1.37–6.14)^2^	1.49 (0.68–3.26)
	28–31	38 (15.8)	13.7 (7.65–24.5)^3^	2.31 (1.02–5.26)^1^
	25–27	22 (55.0)	47.7 (26.8–84.9)^3^	2.44 (0.90–6.61)
BW, g	> 2500	9 (1.1)		
	1500-2499	23 (2.5)	2.23 (1.04–4.78)^1^	0.95 (0.40–2.25)
	1000-1499	41 (23.8)	21.6 (10.7–43.5)^3^	2.25 (0.83–6.13)
	< 1000	14 (70.0)	63.3 (31.1–128)^3^	3.66 (1.16–11.6)^1^
Cesarean delivery	No	58 (7.5)		
	Yes	29 (2.5)	0.33 (0.21–0.51)^3^	0.53 (0.34–0.83)^2^
5-min Apgar < 7	No	47 (2.6)		
	Yes	40 (24.0)	9.04 (6.12–13.4)^3^	2.73 (1.75–4.26)^3^
Birth defects	No	67 (3.7)		
	Yes	20 (13.0)	3.46 (2.16–5.55)^3^	2.57 (1.59–4.16)^3^
Admitted within 24 h (after birth)	No	8 (2.1)		
	Yes	79 (5.0)	2.35 (1.15–4.82)^1^	0.81 (0.39–1.69)
RDS	No	29 (1.7)		
	Yes	58 (21.2)	12.2 (7.97–18.7)^3^	2.76 (1.65–4.65)^3^
Pneumonia/sepsis	No	20 (1.7)		
	Yes	67 (8.6)	5.02 (3.07–8.20)^3^	0.74 (0.43–1.28)
NEC	No	72 (3.8)		
	Yes	15 (39.5)	10.4 (6.62–16.4)^3^	1.76 (1.03–3.03)^1^
Surfactant	No	50 (2.9)		
	Yes	37 (17.1)	5.88 (3.94–8.78)^3^	0.43 (0.28–0.66)^3^
NIV/MV	No	6 (0.5)		
	Yes	81 (10.5)	20.4 (8.93–46.4)^3^	6.69 (2.07–21.7)^2^

Values are presented as number of deaths (%) and corresponding relative risk (RR) and its 95% confidence intervals (95% CI) by Poisson regression analysis. Multivariable analysis was done by all listed factors in the first column. For definition of all-death see Table 1 legends, note 6; for definition of in-hospital deaths see Table 1 legends, note 5; for other definitions and abbreviations see Table 1 and 3 legends as reference

1, 2 and 3 stands for *P* < 0.05, *P* < 0.01 and *P* < 0.001, respectively

The original article [1] has been updated.
