# Supplementary material for: A birth population-based survey of preterm morbidity and mortality by gestational age
Source: BMC Pregnancy Childbirth. 2021 Apr 10;21:291. doi: 10.1186/s12884-021-03726-4 (PMC8037918; doi:10.1186/s12884-021-03726-4)
Supplement: Supplementary file 1 — Additional file 1. Definitions of pregnancy and perinatal co-morbidities and complications and standard of care at admission and during hospitalization of preterm infants. [file 12884_2021_3726_MOESM1_ESM.docx]

Definitions of pregnancy co-morbidities and complications

Prelabor rupture of membrane was diagnosed on the basis of the patient’s history and physical examination, confirmed by the visualization of amniotic fluid passing from the cervical canal and pooling in the vagina.^23^ Hypertensive disorder of pregnancy included chronic hypertension, new-onset gestational hypertension, preeclampsia, eclampsia and hemolysis, elevated liver enzymes and low platelet count syndrome.^24^ Gestational diabetes mellitus is a condition in which carbohydrate intolerance develops during pregnancy, including pre-pregnancy diabetes mellitus.^25^ Anemia in pregnancy was defined as a hemoglobin concentration less than 80 g/L.^26^

Definitions of perinatal co-morbidities and complications

Intraventricular hemorrhage (IVH) was diagnosed within 7 postnatal day (PND) by cranial ultrasound or computed tomography, grades III and IV according to the modern grading system were included.^27^ Respiratory distress syndrome (RDS) was diagnosed by clinical evidence of respiratory difficulties, hypoxemia and chest X-ray appearances.^28^ Pneumonia / sepsis included chest X-ray evidence, positive culture and clinical confirmation excluding aspiration. Hypoglycemia was diagnosed according to clinical report defined as glucose < 25 mg/dl within postnatal 4 hours, or < 35 mg/dl within postnatal 4-24 hours, or < 40 mg/dl beyond 1^st^ PND by American Academy of Pediatrics (AAP) in 2011.^29^ Patent ductus arteriosus only included those requiring oral drugs or surgery for hemodynamical instability. Hyperbilirubinemia referred to those received phototherapy or exchange transfusion according to the guideline by AAP.^30^ Bronchopulmonary dysplasia (BPD) was considered only for those of moderate to severe BPD, defined as requiring supplemental oxygen at 36 weeks’ post menstrual age or at discharge, or continuous positive airway pressure / mechanical ventilation.^31^ Necrotizing enterocolitis (NEC) diagnosis required > 1 abdominal sign and > 1 radiographic finding. Stage II and III of NEC by Bell’s criteria were included.^32^ Retinopathy of prematurity was diagnosed according to policy statement by AAP, and > stage 1 were included.^33^ Encephalopathy included hypoxic ischemia encephalopathy, periventricular leukomalacia, acute bilirubin encephalopathy / kernicterus, and cerebral lesions due to hypoglycemia.^34^ Air leak included pneumothorax, pneumomediastinum and pneumopericardium.

Standard of care at admission and during hospitalization.

For those diagnosed with mild morbidities only, they were admitted to neonatal wards.^7,28^ For those diagnosed with moderate and severe morbidities, they were admitted to neonatal intensive care unit (NICU)^7,28,38^; and critical care was provided to those with severe perinatal and neonatal morbidities, such as Apgar score at 5 min < 3, gestational age (GA) < 28 week, BW < 1,000 g, IVH grade III-IV,^38^ RDS and pneumonia / sepsis requiring intratracheal ventilation, hemodynamic instability requiring vasopressors or inotropes, or complicated with pulmonary hypertension,^28^ hyperbilirubinemia requiring exchange transfusion, moderate-to-severe BPD, NEC stage II-III, encephalopathy requiring robust monitoring and intervention, severe neurological disorders, and surgical interventions, etc.^38^
