# Supplementary material for: A birth population-based survey of preterm morbidity and mortality by gestational age
Source: BMC Pregnancy Childbirth. 2021 Apr 10;21:291. doi: 10.1186/s12884-021-03726-4 (PMC8037918; doi:10.1186/s12884-021-03726-4)
Supplement: Supplementary file 2 — Additional file 2. A full list of members and their affiliations of Huai’an Perinatal-Neonatal study group. [file 12884_2021_3726_MOESM2_ESM.docx]

**A full list of members and their affiliations of Huai’an Perinatal-Neonatal study group**

Hongni Yue^3,4^, Xiaoqiong Li^2^, Tingting Qi^2^, Zhaojun Pan^3^, Guofang Zheng^3^, Xiaoqin Zhu^4^, Hui Wang^4^, Weijie Ding^4^, Muling Zhang^5^, Zhaofang Tian^6^, Honghua Guan^7^, Juan Yang^8^, Shouzhong Li^9^, Keyan Zhu^10^, Chunhong Tang^11^, Maotian Dong^12^, Yaodong Yin^13^, Haijun Wang^14^, Xihui Cao^15^, Jianya Zhang^16^, Hong Liu^17^, Zhou Xu^18^, Cui Gao^19^, Yongbo Heng^20^, Jie Sui^21^, Juan Li^22^, Xia Wu^23^, Chunhua Zhang^24^, Xueya Shen^25^, Jianqin Ding^26^, Juping Xia^27^, Feng Ni^28^, Xueyun Chen^29^, Zhaoxia Wang^30^.

2. Department of Obstetrics, Huai’an Women and Children’s Hospital, Huai’an, Jiangsu, China;

3. Department of Neonatology, Huai’an Women and Children’s Hospital, Huai’an, Jiangsu, China;

4. Unit of Population Health Information, Huai’an Women and Children’s Hospital, Huai’an, Jiangsu, China;

5. Department of Obstetrics, Huai’an First General Hospital, Huai’an, Jiangsu, China;

6. Department of Neonatology, Huai’an First General Hospital, Huai’an, Jiangsu, China;

7. Department of Obstetrics, Huai’an Second General Hospital, Huai’an, Jiangsu, China;

8. Department of Neonatology, Huai’an Second General Hospital, Huai’an, Jiangsu, China;

9. Department of Obstetrics, Huaiyin District Hospital, Huai’an, Jiangsu, China;

10. Department of Neonatology, Huaiyin District Hospital, Huai’an, Jiangsu, China;

11. Department of Obstetrics, Chuzhou District Hospital, Huai’an, Jiangsu, China;

12. Department of Neonatology, Chuzhou District Hospital, Huai’an, Jiangsu, China;

13. Department of Obstetrics, Lianshui County Hospital, Huai’an, Jiangsu, China;

14. Department of Neonatology, Lianshui County Hospital, Huai’an, Jiangsu, China;

15. Department of Obstetrics, Xuyi County Hospital, Huai’an, Jiangsu, China;

16. Department of Neonatology, Xuyi County Hospital, Huai’an, Jiangsu, China;

17. Department of Obstetrics, Hongze County Hospital, Huai’an, Jiangsu, China;

18. Department of Neonatology, Hongze County Hospital, Huai’an, Jiangsu, China;

19. Department of Obstetrics, Jinhu County Hospital, Huai’an, Jiangsu, China;

20. Department of Neonatology, Jinhu County Hospital, Huai’an, Jiangsu, China;

21. Unit of Population Health Information, Qingjiangpu District Women and Children’s Hospital, Huai’an, Jiangsu, China;

22. Department of Obstetrics, Huaiyin District Women and Children’s Hospital, Huai’an, Jiangsu, China;

23. Unit of Population Health Information, Huaiyin District Women and Children’s Hospital, Huai’an, Jiangsu, China;

24. Department of Obstetrics, Huai’an District Women and Children’s Hospital, Huai’an, Jiangsu, China;

25. Unit of Population Health Information, Huai’an District Women and Children’s Hospital, Huai’an, Jiangsu, China;

26. Department of Obstetrics, Lianshui County Women and Children’s Hospital, Huai’an, Jiangsu, China;

27. Unit of Population Health Information, Lianshui County Women and Children’s Hospital, Huai’an, Jiangsu, China;

28. Departments of Obstetrics and Population Health Information, Xuyi County Women and Children’s Hospital, Huai’an, Jiangsu, China;

29. Department of Obstetrics, Xuyi County Maba Central Health Center, Huai’an, Jiangsu, China;

30. Department of Obstetrics, Lianshui County Third General Hospital, Huai’an, Jiangsu, China.
