# Supplementary material for: A birth population-based survey of preterm morbidity and mortality by gestational age
Source: BMC Pregnancy Childbirth. 2021 Apr 10;21:291. doi: 10.1186/s12884-021-03726-4 (PMC8037918; doi:10.1186/s12884-021-03726-4)
Supplement: Supplementary file 3 — Additional file 3: Table S1. Crude and gestational age-adjusted odds ratio (OR) of death risks of the whole Huai’an region. [file 12884_2021_3726_MOESM3_ESM.docx]

Table S1. Crude and gestational age-adjusted odds ratio (OR) of death risks of the whole Huai’an region.

A. The total preterm births associated all-death risks

_____________________________________________________________________

Variables^1^ Crude OR (95% CI) aOR (95% CI)

_____________________________________________________________________

Born in HWCH 1.11 (0.86-1.44) 0.79 (0.58-1.08)

PROM 0.67 (0.50-0.91)** 0.55 (0.38-0.78)**

HDP 0.87 (0.60-1.25) 1.24 (0.83-1.85)

GDM 1.13 (0.63-2.04) 1.69 (0.86-3.33)

Anemia 0.61 (0.40-0.95)* 0.60 (0.37-0.99)*

ANG 0.50 (0.38-0.67)* ** 0.42 (0.30-0.57)***

Cesarean delivery 0.22 (0.17-0.30)*** 0.38 (0.28-0.53)***

Male 0.82 (0.51-1.34) 0.83 (0.49-1.42)

Multiple births 0.76 (0.54-1.05) 0.67 (0.45-0.99)*

SGA 15.2 (1.80-127.9)*** 2.07 (1.58-7.45)***

AF contamination 5.19 (3.73-7.26)*** 5.21 (3.50-7.77)***

5-min Apgar < 7 73.5 (51.3-105.3)*** 25.7 (17.3-38.1)***

Birth defects 7.38 (4.64-11.7)*** 8.27 (4.53-15.2)***

_____________________________________________________________________

B. The hospitalization-associated preterm in-hospital death risks

_____________________________________________________________________

Variables^1^ Crude OR (95% CI) aOR (95% CI)

_____________________________________________________________________

Admitted in HWCH 1.65 (1.05-2.58)* 1.26 (0.77-2.06)

Male 0.61 (0.40-0.95)* 0.52 (0.32-0.84)**

Cesarean delivery 0.31 (0.20-0.49)** 0.58 (0.35-0.96)*

5-min Apgar < 7 11.6 (7.32-18.3)*** 3.70 (2.23-6.13)***

Birth defects 3.83 (2.26-6.51)*** 4.08 (2.14-7.77)***

Admitted within 24 h (after birth) 2.42 (1.16-5.05)* 1.12 (0.42-1.98)

RDS 15.2 (9.55-24.3)*** 4.34 (2.43-7.73)***

Pneumonia/sepsis 5.40 (3.25-8.97)*** 1.91 (1.09-3.32)*

NEC 16.6 (8.30-33.1)*** 6.99 (2.96-16.5)***

Surfactant 6.88 (4.38-10.8)*** 1.14 (0.66-1.96)

NIV/MV 22.6 (9.82-52.1)*** 8.99 (3.62-22.3)***

_____________________________________________________________________

Values are presented as crude and adjusted odds ratio (OR) for gestational age strata with Mantel-Haenszel Chi-square test and its 95% confidence interval (95% CI). For definition of all-death see Table 1 legends, note 6; for definition of in-hospital deaths see Table 1 legends, note 5; for other definitions and abbreviations see Table 1 and 3 legends as reference.

1. Taking null of the variable as reference.

*, ** and *** stands for *P*<0.05, *P*<0.01 and *P*<0.001, respectively.
